# Pendulating—A grounded theory explaining patients’ behavior shortly after having a leg amputated due to vascular disease

**DOI:** 10.3402/qhw.v11.32739

**Published:** 2016-09-16

**Authors:** Ulla Riis Madsen, Ami Hommel, Carina Bååth, Connie Bøttcher Berthelsen

**Affiliations:** 1 Department of Orthopedic Surgery, Slagelse & Holbæk Hospital, Region Sjaelland, Denmark; 2 Faculty of Health, Department of Health Sciences, Lund University, Lund, Sweden; 3 Faculty of Health, Department of Health Sciences, Karlstad University, Karlstad, Sweden; 4 Institute of Health, Section of Nursing, Aarhus University, Aarhus, Denmark

**Keywords:** Adaption, coping, dysvascular amputees, grounded theory, life situation, orthopedic nursing, pendulating, post-operative care, psycho-social needs, quality of life

## Abstract

**Introduction:**

Although the group of vascular leg amputated patients constitutes some of the most vulnerable and frail on the orthopedic wards, previous research of amputated patients has focused on patients attending gait training in rehabilitation facilities leaving the patient experience shortly after surgery unexplored. Understanding patients’ behavior shortly after amputation could inform health professionals in regard to how these vulnerable patients’ needs at hospital can be met as well as how to plan for care post-discharge.

**Aim:**

To construct a grounded theory (GT) explaining patients’ behavior shortly after having a leg amputated as a result of vascular disease.

**Method:**

In line with constructivist GT methodology, data from ethnographic observations and interviews were simultaneously collected and analyzed using the constant comparative method covering the patients’ experiences during the first 4 weeks post-surgery. Data collection was guided by theoretical sampling and comprised 11 patients. A GT was constructed.

**Results:**

Patients went through a three-phased process as they realized they were experiencing a life-changing event. The first phase was “Losing control” and comprised the sub-categories “Being overwhelmed” and “Facing dependency.” The second phase was “Digesting the shock” and comprised the sub-categories “Swallowing the life-changing decision,” “Detecting the amputated body” and “Struggling dualism.” The third phase was “Regaining control” and comprised the sub-categories “Managing consequences” and “Building-up hope and self-motivation.” “Pendulating” was identified as the core category describing the general pattern of behavior and illustrated how patients were swinging both cognitively and emotionally throughout the process.

**Conclusion:**

The theory of “Pendulating” offers a tool to understand the amputated patients’ behavior and underlying concerns and to recognize where they are in the process. Concepts from the theory could be used by health professionals who support patients coping with the situation by offering terms to express and recognize patients’ reactions.

Patients’ need of care immediately after leg amputation due to vascular disease has only been investigated briefly and post-discharge (Fleury, Salih, & Peel, 2013). Studies indicate that health professionals focus on physical and practical issues, but often leave patients alone with emotional and existential suffering (Liu, Williams, Liu, & Chien, 2010; Norlyk, Martinsen, & Kjaer-Petersen, 2013). The majority of major leg amputations performed in the western world is caused by vascular disease (Global Lower Extremity Amputation Study Group, 2000) and are often preceded by a long, troublesome period of wounds, diagnostic trajectories (Denmark's National Board of Health, Sundhedsstyrelsen, 2011; Ragnarson Tennvall & Apelqvist, 2000), vascular surgery, and pain (Schoppen et al., 2003). The amputation is often performed sub-acutely with the aim of ensuring survival for patients who have infections or acute embolus as well as to relieve pain and ensure the best possible level of function for patients (Game, 2012) as they face remarkable physical challenges while recovering from surgery (Back-Pettersson & Bjorkelund, 2005). Patients who have leg amputation due to vascular disease are characterized by high age (Global Lower Extremity Amputation Study Group, 2000), multi co-morbidity (Fortington, Rommers, Geertzen, Postema, & Dijkstra, 2012; Kristensen, Holm, Kirketerp-Moller, Krasheninnikoff, & Gebuhr, 2012), and low survival prognosis (Fortington et al., 2013; Kristensen et al., 2012).

Losing a leg presents an array of physical, emotional, and social challenges for patients (Horgan & MacLachlan, 2004). Several studies have documented that patients struggle with higher levels of anxiety, depression, restricted mobility, and social isolation (Briggs, 2007; Horgan & MacLachlan, 2004). Furthermore, themes of low self-esteem, changes in self, and a struggle to accept a new identity as disabled (Senra, Oliveira, Leal, & Vieira, 2012) are described as dominant among amputees in the months following amputation. Patients also deal with a sense of grief, loss, and shock (Hanley et al., 2004). Previous studies are mostly cross-sectional and include selected populations of patients attending gait-training at rehabilitation facilities (Briggs, 2007; Fleury et al., 2013). Consequently, the psychosocial challenges among patients not attending gait-training as well as the immediate reactions to amputation have yet to be investigated (Horgan & MacLachlan, 2004). Liu et al. (2010) investigated the lived experience after amputation among 22 Taiwanese amputees attending gait-training 2 months post-discharge and found participants reported suffering in the physical and psychosocial realms and felt strongly that their lives had completely changed. Although they appreciated the amputation intellectually, they simultaneously struggled to accept the decision emotionally and found professional help primarily directed on physical and practical aspects of the amputation even though their focus was on coping with fear and anxiety. In a Danish study performed 1–5 months post-discharge, Norlyk et al. (2013) found losing a leg to be a radical and existential upheaval which restricts patients’ lifestyles and changes their lifeworld dramatically. Restrictions to lifeworld were related to a sense of great loss as well as hope of regaining lost territory and personal independence. It is interesting to note that participants were not always supported by health professionals during this process.

Previous research indicates a gap in current care regarding support of leg amputation patients’ transition towards life as physically impaired and leaves the patient experience shortly after surgery unexplored. Understanding patients’ behavior shortly after amputation could inform health professionals in regard to how these vulnerable patients’ needs at hospital can be met as well as how to plan for care post-discharge which includes emotional and existential dimensions.

## The study

### Aim

To construct a grounded theory (GT) explaining patients’ behavior shortly after having a leg amputated because of vascular disease.

Research question: What is the main concern of patients shortly after having a leg amputated and how do they resolve it?

### Design

A constructivist GT approach was used (Charmaz, 2014) which brought subjectivity into view and assumes that people, including researchers, are constructing the realities in which they participate. Constructivist grounded theorists aim for abstract understanding of studied life and view their analysis as located in time, place, and the situation of inquiry (Charmaz, 2014).

### Setting

The study was performed from April 2013 to January 2014 in orthopedic wards of two regional hospitals in rural Denmark. These hospitals perform 100 leg amputations annually. The care is organized in clinical pathways and mean length-of-stay in hospital after amputation is 11 days.

### Participants

Initial recruitment of participants was based on the researchers wish to investigate concerns and behavior among the heterogeneous population of patients who experienced leg amputation due to vascular disease for the first time. This meant that the researchers did not limit themselves to patients who could participate in prosthesis training. Thus, the first two patients who were Danish-speaking and did not have a diagnosis of dementia were included based on accessibility. Further recruitment was guided by the principles of theoretical sampling (Charmaz, 2014) and choices were made about where to look for data that could expand the emerging categories and concepts. As the researchers’ focus was the patients’ immediate reactions, and it was found early in the study that observing the patients in hospital gave insight not available when the participants had rationalized their experiences after discharge, it was decided to continue recruiting patients experiencing their first leg amputation based on accessibility. It was also decided that the same data collection process would be used for all participants.

In all, eight men and three women were recruited within 3 days of unilateral leg amputation due to vascular disease. Age range of participants was 45–84. Two patients only participated in-hospital: one withdrew his consent and one was re-amputated before the after-discharge interview. Six participants had undergone below-knee amputation, one was amputated through the knee and four above the knee. All had at least one comorbidity. Six participants lived with their spouse and the rest lived alone. All participants were retired. All but one was discharged to their former independent living situation with the latter being released to a temporary nursing home.

### Data collection

Data collection, analysis, and coding were performed simultaneously. By constant comparison of concepts and incidents, data emerged while memos were written (Charmaz, 2014). The study was designed to gain insight into the participants’ views, feelings, intentions, and actions shortly after amputation. Attempting not to interfere with the process under study and recognizing the vulnerability of these patients, ethnographic observations were performed during hospital stay and in-depth interviews given 2 weeks post-discharge. This was performed to compare the behavior observed with the narratives told by the participants at interviews and, thereby, expand understanding as recommended by Charmaz (2014).

All data were collected by the first author who met each participant on four occasions during the time he or she was admitted to hospital as well as at their homes 2 weeks post-discharge. The first occasion was when consent was obtained between days 1 and 3. When the project was presented with the phrase “I'm investigating what people's concerns are when they have had a leg amputated,” all participants had a narrative that told what was on their mind. Immediately after, notes of observations were written and stored on a computer.

On the second occasion, non-participant observations (Spradley, 2008) were performed during the bedside meeting 3–5 days after amputation where, according to the clinical pathway, the physician, nurse and physiotherapist were to evaluate whether the patient was suitable for prosthesis fitting as well as make plans to discharge the patient. The observer sat in the back of the room to avoid interfering with ongoing interaction. Field notes were taken during the observations with full observation notes being written immediately after. Each observation lasted 20–45 min.

The third occasion occurred later the same day when participants were encouraged to further assess and explore opinions and feelings that arose from meeting with their healthcare providers. On the fourth occasion, the participants were approached the day before discharge to arrange an interview appointment 2 weeks later. This informal interview started where the participant was at that point. Some hardly remembered being part of a research project while others picked up the conversation from the last meeting. Again, notes of observation were written immediately after the interviews and stored on a computer.

In-depth interviews (Charmaz, 2014) were performed at the patients’ homes 2 weeks post-discharge. Three of the participants wanted their spouses present to support their memory. An interview guide with open-ended questions was developed to help the researcher cover the concepts of interest and was introduced with the statement, “I am interested in your experiences and concerns while you were hospitalized to have your leg amputated.” The interview was started with the question, “Would you please start telling me what led to the amputation?” followed by questions about experiences and concerns during hospital stay. Incidents and concepts from the in-hospital observations were brought forward to explore the opinions and feelings associated with them. Additionally, all participants talked about getting home and their present concerns. The interview guide was customized from interview to interview as analysis developed and was, thus, congruent to theoretical sampling (Charmaz, 2014). Interviews lasted between 58 and 65 min and were digitally recorded and transcribed verbatim by the first author. Data consist of field notes from 30 informal meetings, 10 non-participant observations and transcripts of nine in-depth interviews. All of this combined information is treated as a single lot of data in analysis. Analysis was performed by the first author and supervised by the last author who read all coded data.

### Data analysis

Transcriptions from observations, interviews and memos were initially coded line-by-line looking for behavior related to the research question, “What is the main concern of patients shortly after having a leg amputated and how do they resolve it?” Initial codes were compared while looking for patterns in data and constructing early concepts. Further data were collected, coded line-by-line and compared until seven categories could be constructed (Table I) along with the patients’ main concern: “How do I manage my life after having lost a leg?” Further data collection was based on the seven categories according to theoretical sampling and focused coding was conducted (Charmaz, 2014) to delimit data collection only to the relevant categories. Theoretical sampling ceased when these categories were saturated and further data collection did not contribute new knowledge to the emergent theory. Through continually and systematically comparing categories with concepts while writing memos, analysis was brought to a higher level of abstraction which revealed properties and a range of the emergent categories. Finally the “puzzle” was put together by explaining the behavior of the patients as reactions in a three-phased process where they realize they are experiencing a life-changing event. Eventually, the core category of Pendulating emerged to describe the general pattern of behavior throughout the process. The presented findings are, in line with constructivist GT (Charmaz, 2014), the product of this analytic process and do not distinguish between the various types of data the analysis is based on.

**Table I:** The process of analysis and coding.

Code	Category	Phase
Having extensive thoughts	Being overwhelmed	Losing control
Having fragmented memory		
Letting things happen		
Defending and protecting one self		
Surrendering	Facing dependency	
Escaping		
Limiting the consequences		
Hoping to get a prosthesis		

Seeking confirmation	Swallowing the life-changing event	Digesting the shock
Torturing oneself with mental pictures		
Facing lethal consequences		
Relating to surgeon	Detecting the amputated body	
Telling about body experiences		
Awareness of physical appearance		
Having a limiting picture of ability as amputated		
Feeling relieved and yet frustrated	Struggling dualism	
Being torn between desire and reality		
Losing courage		

Not knowing what to expect	Managing consequences	Regaining control
Downscaling expectations and compromising to solve practicalities		
Knowing adapting takes time		
Counting positive signs	Building up hope and self-motivation	
Comparing with other (worst case)		
Sorting bad memories of		
Prioritizing functioning over feelings		

### Ethical considerations

The study was approved by the Danish Data Protecting Agency (Region Sjaellands j.nr. 12-000179) and has been presented to the Regional Ethics Committee whose secretariat did not find the project notifiable under Danish law (Region Sjaelland j.nr. 12-000660). In accordance with the basic principles for research given in the Helsinki Declaration and the Northern Nurses’ Federation (2003), the patients received written and verbal information about the purpose of the study, were informed of their right to withdraw and were advised about the confidentiality of the data given before giving their written consent.

## Findings

**Figure 1:**
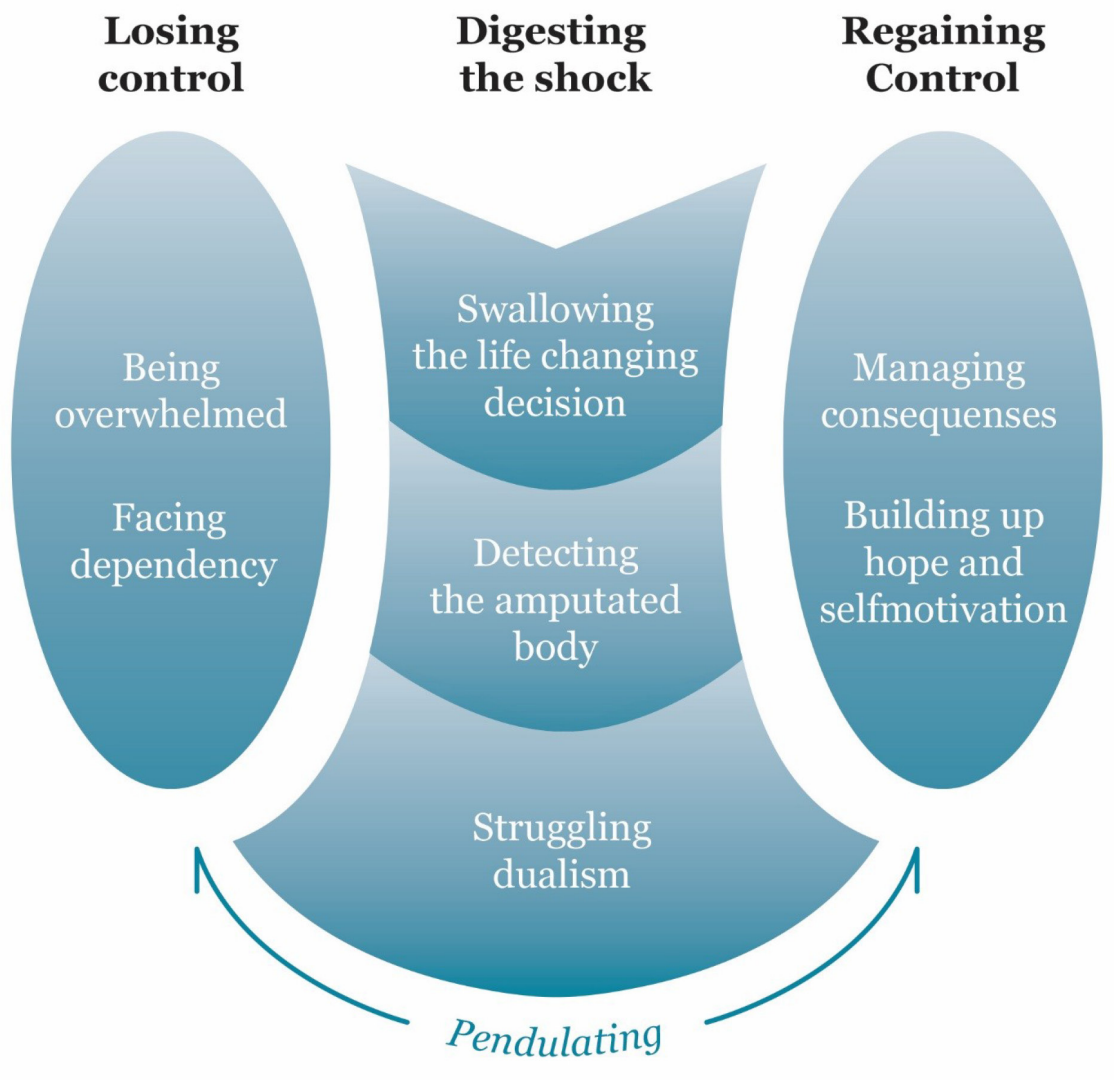
The substantive theory of Pendulating. The figure is illustrating the three-phased process that patients go through shortly after having a leg amputated while realizing they are experiencing a life-changing event.

The GT of “Pendulating” (visualized in Figure 1) was constructed to explain the patients’ behavior shortly after having a leg amputated due to vascular disease. We found that the patients went through a three-phased process as they realized they were experiencing a life-changing event. “Pendulating” was identified as the core category describing the general pattern of behavior and illustrates how the patients swung both cognitively and emotionally throughout the process. The patients’ main concern was “How do I manage my life after having lost a leg?” The three phases of the process are labeled “Losing control,” “Digesting the shock,” and “Regaining control.”

### Losing control

Losing control indicated the first phase of the process and comprised the sub-categories “Being overwhelmed” and “Facing dependency.” One put it this way:

I think that even though you don′t want to admit it, you have got a fright. There is something inside you which you have never experienced before, and you think thousands of thoughts—what will happen and how will I manage and things like that. But you do not hold onto any of those thoughts because you are simply just not able to. (Male 84 years, interview)

*Being overwhelmed* explained how patients reacted as they were put out of action by medication, experienced deteriorated health and dealt with emotional shock. Experiencing a period of fragmented memory or confusion was common. This experience was unpleasant and resulted in fear that the patient would not regain clarity of mind. The patients were aware that they needed help to make plans and see the bigger picture and sought this help from relatives.

When interacting with health professionals, the patients took a passive role, answered questions politely and calmly received advice and information. In spite of this, they were not met with appropriate patience from professionals as the patients, in their overwhelmed state, tried to find words to communicate. Some protected themselves by choosing who they trusted and wanted to relate to which resulted in them communicating only the absolute necessities to the rest of the professionals.

This period of “Being overwhelmed” lasted a few days or continued for weeks.

*Facing dependency* explained how patients reacted to the need for assistance and personal aids necessitated by the amputation. Their actions were characterized by ignorance and uncertainty about the future. How they thought of themselves was influenced by their own stigmatized view of worth and ability of disabled people. As one said:

Well now I am handicapped. Now I am done. (Woman 82, interview)

These thoughts were often not spoken aloud but were part of the “thousand thoughts” described previously. The patients repeatedly spoke of specific issues, often related to participation in future social life, without really knowing what to expect realistically. Some thought they had to give up all independent mobilization. Others compared themselves with amputated elite soldiers who run on two prostheses.

Getting a prosthesis was, at this early stage, the only obvious and desirable solution for getting mobility back, and the process of prosthesis fitting was envisioned as a passive process comparable to fitting a pair of boots. In contrast, the wheelchair became the visual symbol of the undesirable dependency; therefore, the wheelchair was tolerated but not accepted.

Patients who accepted the inevitability of the situation maintained a sense of control by surrendering to their destiny. As one stressed:

I cannot run away if I wanted to. I have no leg to run with. (Male 75, observation)

Surrendering meant downscaling expectations by accepting a lower level of functionality and uncritically accepting the support offered while holding onto modest hopes of regaining mobility, if possible with a prosthesis, but most of all aiming to manage everyday life at home.

Others mentally escaped by thinking of the situation as temporary until they could get a prosthesis and walk again regardless of whether this was a realistic option or not. These patients accepted support and aids unwillingly and wanted to go home as soon as possible as they believed going home would solve many problems and ease recovery.

*Pendulating* at this point of the process described how patients were cognitively and emotionally affected as they lost control in more aspects of their lives. They clung to the pendulum which was in constant motion and swung them in and out of heavy thoughts and from one worry to the next as well as through diverse feelings of injustice, relief, panic and gratefulness.

### Digesting the shock

“Digesting the shock” indicated the second phase of the process and comprised the sub-categories “Swallowing the life-changing decision,” “Detecting the amputated body” and “Struggling dualism.” Having a leg amputated was perceived as a life-changing event to which participants had to adjust.

My life has changed dramatically with this operation. (Woman 78, interview)

*Swallowing the life-changing decision* explains how the patients after surgery processed the shock of deciding to have leg amputation. By accepting the amputation, each patient was aware of his or her responsibility although there was no real choice as the pain or the threat of lethal consequences was unendurable.

I had to accept. Otherwise I could risk dying. (Male 83, interview)

Detailed pictorial descriptions of the situation when the decision was made followed them. Some had experienced the physician as compassionate and empathetic and even though the patients had a hard time accepting the amputation, they found this comforting. Others described how the physician had come in and confronted them with the necessity of the amputation or as one described:

He was downright drooling to take my leg off. (Woman 79, interview)

Despite the recommendation for amputation, stories of having a leg rescued in the past made it harder for participants to be convinced of the inevitability of the present situation. It was important to get confirmation from experts and relatives that the amputation had been the right decision post-surgery in order for the patient to “swallow” the decision. Trust in the relationship with the surgeon was described as crucial.

*Detecting the amputated body* described reactions to the changing body. How patients perceived their body was closely related to function:

Asked how he assesses his health today on a 1–10 scale, he evaluates how his arms, legs and body work. About the legs he says, “I can move at least one leg.” I ask if he cannot move the other leg. “Well yes, but I cannot walk! And I live on the second floor!” (Male 84, observation)

At an individual pace, patients began looking at the stump. Some avoided this for as long as possible as they summoned the courage to be visually confronted with the missing leg. Others had prepared themselves. Experiencing phantom-sensations or phantom-pain made patients fear loss of sanity and talking with the professionals about it was comforting. Social awareness made them hide the amputated leg as they imagined other people looked down on them as handicapped.

*Struggling dualism* explained how opposing emotional reactions caused both frustration and alleviation.

A feeling of relief was common among those who had had unbearable pain or stressful wound trajectories preceding the amputation. This positive emotion sometimes overshadowed the difficulties they were facing; yet at the same time, patients struggled with the consequences. As one said:

Before I had two problems (pain and wound), now I have one. (Male 45, interview)

Others were extremely frustrated by the limitations the missing leg put on their lives. These patients struggled with feelings of confusion as they knew the amputation had saved their lives. Some experienced regret about the decision and tortured themselves with mental pictures of the sawn off leg.

Having days where they lost courage and rejected training caused patients to struggle with their conscience and, looking back, they were grateful for professionals who understood and motivated them anyway.

*Pendulating*, at this point, described how patients processed the shock while they, emotionally and cognitively, were rapidly swinging from one side to the other. Some had a degree of control over the swing when they diverted themselves from heavy thoughts by deliberately thinking positively and thereby swinging the pendulum away from their worries. Others tried to stop the pendulum for a moment (e.g., by refusing to participate in training). At the existential level, themes of justice and guilt were predominant and these themes were difficult to put into words. Somehow, patients digested the shock, a necessary step, before moving forward and regaining control.

### Regaining control

“Regaining control” indicated the third phase of the process and comprised the sub-categories “Managing consequences” and “Building up hope and self-motivation.” Ignorance and uncertainty still marked patients’ actions and there was awareness that adaption would take time and require energy.

It won't help me to look back. I will have to make the best out of the situation. (Male 76, interview)

*Managing consequences* explained how the patients began to regain control when they were capable of hanging onto their thoughts long enough to plan and decide how to handle everyday tasks.

The patients who received help from homecare were grateful for the help but described the situation as living in central station. They struggled with their desire for independence and had to downscale expectations as well as compromise to adjust to the situation. An example of this was when patients were trapped inside because of stairs and doorsteps. Having to sleep separated from a spouse was also pointed out as a painful compromises.

Other patients relied on help from family instead of homecare as help from relatives was considered less invasive. These arrangements raised other questions of being a burden to those relatives and sometimes shifted roles between husband and wife. One described how he forced himself to participate in training in order to be less of a burden to his wife. Another instructed her husband to push her to manage tasks on her own so that she would not end up as a passive invalid sitting in the corner.

*Building up hope and self-motivation* described how patients regained emotional control by focusing on their responsibility for creating a good life despite the missing leg and new dependency. All had been confronted with their mortality and stressed that having a good life was up to themselves. As one said:

… and lovely grandchildren, right, I want to see them growing up. So, I thought, I can live without a leg (tears in her eyes, emotion in voice) and then it is up to me to get a good life… (Woman 82, interview)

Adapting to the situation required even more willpower and strength than patients believed they had. Deliberately thinking positively about the future, downscaling difficulties and problems as well as selectively distorting memories in ways that promoted emotional well-being, made the situation easier to accept and was used, along with diverting oneself with busyness, to create self-motivation.

To build up hope, different signs were attributed positive meaning and as markers of luck. This translated into how lucky they felt about the level of their amputation. The ones who had leg amputation below the knee felt fortunate to have more leg left. Others assessed themselves as being fortunate to have leg amputation above the knee as the risk of re-operation was minor. All thought prosthesis-fitting would be easier. Relatives calling, visiting and helping without being asked made them feel worthwhile to other people.

*Pendulating* at this point of the process, described how the patients were emotionally controlling the situation. The pendulum was now swinging more slowly and was mostly controlled by the patients who pushed themselves away from uncomfortable and undesirable thoughts by deliberately thinking of something positive and occupying their minds with practicalities.

Patients were aware of the existential losses having a leg amputation had caused them. These losses included loss of independency, change of social roles, plans for the future and identity as a walking person among other things. In spite of these losses, patients postponed relating to these existential thoughts until they had digested the shock and regained some control.

## Discussion

This study provides a unique insight into patients’ concerns and reasons for acting as they do immediately after having leg amputation. It shows that having a leg amputated is a life-changing event even for frail patients of all ages who were included in this study. This study illustrates how cognitively and emotionally vulnerable patients are shortly after having a leg amputated, which underpins the moral and ethical obligation to plan and perform care to meet the physical, practical, emotional, and existential needs of these patients.

Not surprisingly, the identified phases of “Losing control” and “Digesting the shock” have similarities with the well-known grief phases of shock and reaction (Cullberg, 2007). This study adds detailed insight into the process specific for patients having lost a leg. It was surprising how little the patients’ struggle in the phase “Losing control” was acknowledged when they were cared for in hospital. While experiencing “Being overwhelmed” and “Facing dependency,” the clinical pathway advised that caregivers discuss rehabilitation goals, prosthesis and other practicalities in order to plan discharge. This was performed without always giving patients’ time to express themselves. Consequently, the patients protected and defended themselves when interacting with professionals and many of the patients’ worries were never communicated. Instead they repeatedly addressed specific issues of more a practical nature and chose the professionals whom they trusted and related to. This meant that patients only communicated what was absolutely necessary with the rest of their healthcare providers.

These findings provide important additional insight into the experience of patients and details what other studies have found about patients experiencing professional help as primarily directed towards the physical and practical aspects after amputation (Liu et al., 2010; Norlyk et al., 2013). Losing control in the acute phase after having a leg amputated is something patients remember long after as a period of suffering (Livingstone, Mortel, & Taylor, 2011; Sjodahl, Gard, & Jarnlo, 2008), and the fact that losing control is a phase all patients have to live through after having a leg amputated underscores the importance of nurses and other professionals being particularly attentive to these patients’ concerns. The same applies to the phase “Digesting the shock” where deciding to have leg amputation triggered a shock that patients had to process afterwards in spite of intellectually accepting the amputation as being for their good. “Swallowing the life-changing decision” seemed easier for those who experienced empathy when amputation was decided and for those who after surgery had the opportunity to discuss with an expert whom they trusted what had led to the necessity of leg amputation. In spite of this, many patients reported not being met with empathy which underscores the necessity for continued work in hospitals to disseminate knowledge and skills among physicians and other health professionals who care for these patients. This includes how to communicate bad news and how to perform follow-up conversations as recommended in the literature (Fallowfield & Jenkins, 2004; Schmidt Mast, Kindlimann, & Langewitz, 2005).

The theory of “Pendulating” offers a tool to understanding patients’ behavior and underlying concerns as well as to recognize where they are in the process. Concepts from the theory could be used by health professionals who support patients coping with the situation by offering terms to express and recognize their reactions.

Understanding the leg-amputated patient's behavior in the acute setting as a chronically ill person experiencing a crisis that potentially threatens his or her identity helps with understanding the scope of suffering when “Facing dependency.” In studies of the lives of chronically ill people, Charmaz (1983) showed that many ill people hold values of independence and individual responsibility; as a result, these patients question their own self-worth and view developing limitations as losses. In this study, the category “Facing dependency” explains how participants reacted to the need for assistance and personal aids. The behavior of the participants’ mentally escaping resembles a category of “Facing dependency” constructed by Charmaz (1991/1997) which explains how many chronically ill people cannot accept dependency, even when foisted upon them, as dependency remains a greater specter than death. Patients may reject anything that they view as a symbol of failing health or a testimony to dependency. This study expands the category by adding that some patients surrender by accepting the inevitability of the situation. They downscale expectations and uncritically accept the support offered. There is a risk that these patients will meet more restrictions than necessary as it does not occur to them to ask for additional aids or support.

The experience of having a leg amputated separates patients from other achieved handicaps as patients believe they will have the possibility of getting a prosthesis whether that is a realistic option or not. Imagining how the missing leg can be replaced with a prosthesis constitutes a symbol of “Regaining independence.” In contrast, symbols of disability, such as the wheelchair, are neglected or rejected in the first few days after amputation as part of coping with the situation. “Facing dependency” underpins the importance of acknowledging and supporting the patient's wish of returning to as normal and independent a life as possible whether they are they younger, older, stronger, or weaker.

This study has several limitations. To increase credibility, data collection was performed in a heterogenic sample of participants by one researcher who achieved intimate familiarity with the setting through a combination of observation and interviews. Analysis in a qualitative study is built upon a subjective process where the researcher is an important actor and preconceived ideas may influence data collection as well as analysis. To minimize this risk, the last author, who also assisted the process of analysis, read all coded data. Two important criteria to evaluate a GT are resonance and usefulness (Charmaz, 2014). These criteria determine whether the theory makes sense to the participants and/or people who share their circumstances. It also helps evaluate if people can use the information in their everyday world. While writing this manuscript, concepts from the theory were presented to more than 40 patients who had experienced leg amputation within the prior 2 weeks as part of another research project. These patients immediately connected to the concepts of “Losing control” and “Digesting the shock.” This indicates resonance and usefulness although further research is needed.

## Conclusion

The substantive theory of “Pendulating” explains the behavior and underlying concerns of patients shortly after leg amputation and is linked to the process of realizing that they are experiencing a life-changing event which has potential life- and identity-threatening consequences. This study offers unique insight into this vulnerable group of patients’ experiences not previously examined and underpins the moral and ethical obligation to plan and perform care to meet the physical, practical, emotional and existential needs of these patients. The theory of “Pendulating” offers a tool to understand the patients’ behavior and underlying concerns and to recognize where they are in the process. Concepts from the theory could be used by health professionals who support patients coping with the situation by offering terms to express and recognize patients’ reactions. Taking the insights from this study into consideration, more research is warranted to test modes of pre- and post-operative care.
